# Tripropyl­ammonium trithio­cyanurate

**DOI:** 10.1107/S1600536810038924

**Published:** 2010-10-13

**Authors:** Yunxia Yang

**Affiliations:** a Key Laboratory of Eco-environment-related Polymer Materials, Ministry of Education, College of Chemistry and Chemical Engineering, Northwest Normal University, Lanzhou 730070, Gansu, People’s Republic of China

## Abstract

In the title compound (systematic name: tripropyl­azanium 2,4,6-tris­ulfanyl­idene­cyclo­hexan-1-ide), (C_3_H_7_)_3_HN^+^·C_3_H_2_N_3_S_3_^−^, one H atom of trithio­cyanuric acid is accepted by tripropyl­amine to form the ammonium ion. Coplanar trithio­cyanurate and tripropyl­ammonium ions [dihedral angle = 82.33 (8)°] form the salt, which is stabilised by various N—H⋯S and N—H⋯N contacts.

## Related literature

For the crystal structures of tetra­phenyl­phospho­nium salts of trithio­cyanuric acid, see: Dean *et al.* (2004 ▶).
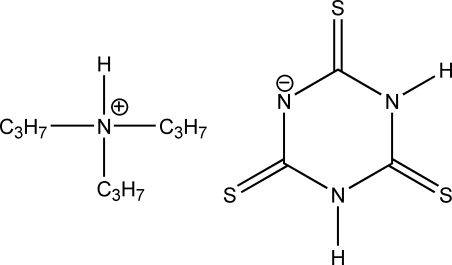


## Experimental

### 

#### Crystal data

C_9_H_22_N^+^·C_3_H_2_N_3_S_3_^−^
*M* *_r_* = 320.53
Orthorhombic, 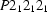

*a* = 8.3677 (5) Å
*b* = 12.8827 (8) Å
*c* = 16.5339 (10) Å
*V* = 1782.33 (19) Å^3^
*Z* = 4
Mo *K*α radiation
μ = 0.41 mm^−1^
*T* = 296 K
0.61 × 0.27 × 0.21 mm

#### Data collection

Bruker APEXII CCD area-detector diffractometer
Absorption correction: multi-scan (*SADABS*; Sheldrick, 1996 ▶) *T*_min_ = 0.788, *T*_max_ = 0.919
5690 measured reflections
3675 independent reflections
3232 reflections with *I* > 2σ(*I*)
*R*_int_ = 0.013

#### Refinement

*R*[*F* ^2^ > 2σ(*F* ^2^)] = 0.035
*wR*(*F* ^2^) = 0.097
*S* = 1.03
3675 reflections
181 parameters
3 restraints
H atoms treated by a mixture of independent and constrained refinement
Δρ_max_ = 0.24 e Å^−3^
Δρ_min_ = −0.20 e Å^−3^
Absolute structure: Flack & Bernardinelli (2000 ▶), 1316 Friedel pairs
Flack parameter: −0.04 (8)

### 

Data collection: *APEX2* (Bruker, 2007 ▶); cell refinement: *SAINT* (Bruker, 2007 ▶); data reduction: *SAINT*; program(s) used to solve structure: *SHELXS97* (Sheldrick, 2008 ▶); program(s) used to refine structure: *SHELXL97* (Sheldrick, 2008 ▶); molecular graphics: *SHELXTL* (Sheldrick, 2008 ▶); software used to prepare material for publication: *SHELXL97* and *publCIF* (Westrip, 2010 ▶).

## Supplementary Material

Crystal structure: contains datablocks I, global. DOI: 10.1107/S1600536810038924/rn2070sup1.cif

Structure factors: contains datablocks I. DOI: 10.1107/S1600536810038924/rn2070Isup2.hkl

Additional supplementary materials:  crystallographic information; 3D view; checkCIF report

## Figures and Tables

**Table 1:** Hydrogen-bond geometry (Å, °)

*D*—H⋯*A*	*D*—H	H⋯*A*	*D*⋯*A*	*D*—H⋯*A*
N4—H4⋯N3	0.93 (1)	1.95 (1)	2.867 (2)	172 (3)
N1—H1⋯S2^i^	0.92 (1)	2.51 (1)	3.4037 (17)	167 (2)
N2—H2⋯S1^ii^	0.91 (1)	2.39 (1)	3.2911 (17)	170 (2)

Symmetry codes: (i) 
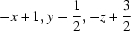
; (ii) 
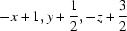
.

**Fig. 1.**
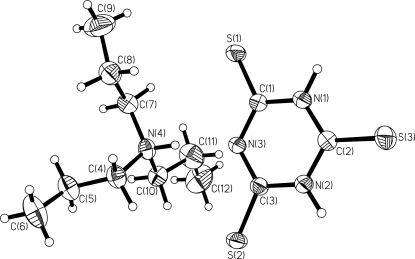
Thermal ellipsoid plot of the title compound at the 30% probability level; hydrogen atoms are drawn as spheres of arbitrary radius.

**Fig. 2.**
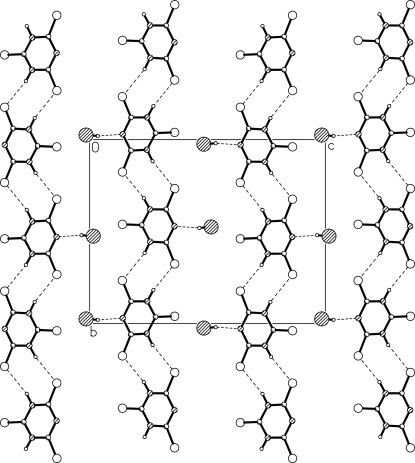
Hydrogen bond pattern in the crystal structure of the title compound; all hydrogen atoms bonded to carbon and carbon atoms of the tripropylammonium cation are omitted for clarity and the cations are represented with the hatched spheres.

## supplementary crystallographic information

### Comment

Trithiocyanuric acid, which can be regarded as the polymer of three thiourea molecules, tends to form various hydrogen bonds with its hydrogen-bond donor and acceptor (Dean *et al.* 2004). Here we reported the cocrystal of the tripropylammonium cation and trithiocyanurate with a molar ratio of 1:1, (C_3_H_7_)_3_HN^+^.C_3_H_2_N_3_S_3_^-^. In this structure, the independent planar trithiocyanurate anion only form a pair of N—H···S hydrogen bonds, of which N···S distances are 3.404 (2) Å and 3.291 (2) Å and the corresponding angles 166.6° and 169.7°, to generate the hydrogen-bonded ribbons along the *b* axis, and these ribbons which are translated by 2_1_ rotation axis are orderly arranged almost along the (101) and (101)directions. Subsequently, the central N—H group of the ammonium cation can form an N—H···N donor hydrogen bond (N···N distance is 2.867 (2) Å and the related angle is 172.5°) with one of the nitrogen atom located in the trithiocyanurate to generate the final stable cocrystal.

### Experimental

Trithiocyanuric acid (0.044 g, 0.25 mmol) was dissolved in a water-ethanol (1:2 *v*/*v*) mixture and tripropylamine was added to neutralize the acid. Colorless block crystals formed after several weeks.

### Refinement

All hydrogen atoms bonded to carbon were introduced to idealized positions and allowed to ride on their parent atoms. Hydrogen atoms bonded to nitrogen were located in difference Fourier syntheses with N—H distance of 0.93 Å.

### Crystal data

**Table:**

C_9_H_22_N^+^·C_3_H_2_N_3_S_3_^−^	*F*(000) = 688
*M**_r_* = 320.53	*D*_x_ = 1.195 Mg m^−^^3^
Orthorhombic, *P*2_1_2_1_2_1_	Mo *K*α radiation, λ = 0.71073 Å
*a* = 8.3677 (5) Å	µ = 0.41 mm^−^^1^
*b* = 12.8827 (8) Å	*T* = 296 K
*c* = 16.5339 (10) Å	Block, colorless
*V* = 1782.33 (19) Å^3^	0.61 × 0.27 × 0.21 mm
*Z* = 4	

### Data collection

**Table:**

Bruker APEXII CCD area-detector diffractometer	3675 independent reflections
Radiation source: fine-focus sealed tube	3232 reflections with *I* > 2σ(*I*)
graphite	*R*_int_ = 0.013
phi and ω scans	θ_max_ = 27.6°, θ_min_ = 2.7°
Absorption correction: multi-scan (*SADABS*; Sheldrick, 1996)	*h* = −4→10
*T*_min_ = 0.788, *T*_max_ = 0.919	*k* = −16→16
5690 measured reflections	*l* = −17→19

### Refinement

**Table:**

Refinement on *F*^2^	Secondary atom site location: difference Fourier map
Least-squares matrix: full	Hydrogen site location: inferred from neighbouring sites
*R*[*F*^2^ > 2σ(*F*^2^)] = 0.035	H atoms treated by a mixture of independent and constrained refinement
*wR*(*F*^2^) = 0.097	*w* = 1/[σ^2^(*F*_o_^2^) + (0.057*P*)^2^ + 0.1498*P*] where *P* = (*F*_o_^2^ + 2*F*_c_^2^)/3
*S* = 1.03	(Δ/σ)_max_ < 0.001
3675 reflections	Δρ_max_ = 0.24 e Å^−^^3^
181 parameters	Δρ_min_ = −0.20 e Å^−^^3^
3 restraints	Absolute structure: Flack & Bernardinelli (2000), 1316 Friedel pairs
Primary atom site location: structure-invariant direct methods	Flack parameter: −0.04 (8)

### Special details

**Table:**

Geometry. All e.s.d.'s (except the e.s.d. in the dihedral angle between two l.s. planes) are estimated using the full covariance matrix. The cell e.s.d.'s are taken into account individually in the estimation of e.s.d.'s in distances, angles and torsion angles; correlations between e.s.d.'s in cell parameters are only used when they are defined by crystal symmetry. An approximate (isotropic) treatment of cell e.s.d.'s is used for estimating e.s.d.'s involving l.s. planes.
Refinement. Refinement of *F*^2^ against ALL reflections. The weighted *R*-factor *wR* and goodness of fit *S* are based on *F*^2^, conventional *R*-factors *R* are based on *F*, with *F* set to zero for negative *F*^2^. The threshold expression of *F*^2^ > σ(*F*^2^) is used only for calculating *R*-factors(gt) *etc*. and is not relevant to the choice of reflections for refinement. *R*-factors based on *F*^2^ are statistically about twice as large as those based on *F*, and *R*- factors based on ALL data will be even larger.

### Fractional atomic coordinates and isotropic or equivalent isotropic displacement parameters (Å^2^)

**Table:**

	*x*	*y*	*z*	*U*_iso_*/*U*_eq_	
N4	0.25770 (18)	0.02494 (16)	0.48456 (11)	0.0513 (4)	
H4	0.307 (3)	0.020 (2)	0.5345 (9)	0.077*	
C4	0.3656 (3)	0.0945 (2)	0.43528 (14)	0.0603 (6)	
H4A	0.3771	0.1602	0.4634	0.072*	
H4B	0.4706	0.0629	0.4324	0.072*	
C5	0.3087 (3)	0.1159 (2)	0.35052 (17)	0.0726 (7)	
H5A	0.2116	0.1570	0.3525	0.087*	
H5B	0.2843	0.0509	0.3237	0.087*	
C7	0.2386 (3)	−0.0818 (2)	0.44928 (15)	0.0596 (5)	
H7A	0.1680	−0.0775	0.4029	0.072*	
H7B	0.1872	−0.1259	0.4891	0.072*	
C8	0.3920 (3)	−0.1327 (2)	0.42319 (19)	0.0756 (7)	
H8A	0.4343	−0.0967	0.3763	0.091*	
H8B	0.4700	−0.1275	0.4664	0.091*	
C9	0.3658 (6)	−0.2448 (3)	0.4026 (3)	0.1228 (14)	
H9A	0.4652	−0.2754	0.3862	0.184*	
H9B	0.2899	−0.2500	0.3593	0.184*	
H9C	0.3255	−0.2807	0.4492	0.184*	
C10	0.0991 (2)	0.0762 (2)	0.50164 (16)	0.0605 (5)	
H10A	0.1181	0.1469	0.5194	0.073*	
H10B	0.0380	0.0794	0.4518	0.073*	
C11	0.0026 (4)	0.0216 (3)	0.5642 (2)	0.0995 (11)	
H11A	−0.0111	−0.0501	0.5476	0.119*	
H11B	0.0620	0.0215	0.6145	0.119*	
C12	−0.1564 (4)	0.0668 (4)	0.5792 (3)	0.1253 (15)	
H12A	−0.2104	0.0272	0.6201	0.188*	
H12B	−0.2178	0.0655	0.5302	0.188*	
H12C	−0.1446	0.1373	0.5972	0.188*	
C6	0.4346 (4)	0.1736 (3)	0.3029 (2)	0.1079 (12)	
H6A	0.3962	0.1865	0.2492	0.162*	
H6B	0.5302	0.1325	0.3003	0.162*	
H6C	0.4576	0.2385	0.3291	0.162*	
N1	0.5530 (2)	−0.05504 (13)	0.74298 (10)	0.0468 (4)	
H1	0.579 (3)	−0.1170 (12)	0.7667 (15)	0.070*	
N2	0.5353 (2)	0.12080 (13)	0.74422 (11)	0.0456 (4)	
H2	0.556 (3)	0.1813 (12)	0.7704 (14)	0.068*	
N3	0.40788 (18)	0.02890 (11)	0.64040 (10)	0.0449 (3)	
S1	0.40179 (9)	−0.17606 (4)	0.63935 (4)	0.06707 (19)	
C1	0.4565 (2)	−0.05976 (15)	0.67496 (12)	0.0451 (4)	
S2	0.37526 (8)	0.23360 (4)	0.63897 (4)	0.06029 (17)	
C2	0.5970 (2)	0.03521 (15)	0.77943 (11)	0.0433 (4)	
S3	0.71163 (7)	0.03940 (5)	0.86074 (4)	0.06204 (17)	
C3	0.4425 (2)	0.12015 (15)	0.67552 (13)	0.0433 (4)	

### Atomic displacement parameters (Å^2^)

**Table:**

	*U*^11^	*U*^22^	*U*^33^	*U*^12^	*U*^13^	*U*^23^
N4	0.0444 (8)	0.0640 (11)	0.0455 (10)	−0.0042 (8)	−0.0042 (7)	0.0042 (9)
C4	0.0544 (12)	0.0713 (15)	0.0551 (14)	−0.0120 (11)	−0.0025 (10)	0.0083 (11)
C5	0.0656 (15)	0.0872 (18)	0.0652 (17)	−0.0049 (12)	−0.0052 (13)	0.0186 (14)
C7	0.0542 (12)	0.0604 (14)	0.0644 (14)	−0.0008 (10)	−0.0020 (10)	0.0050 (12)
C8	0.0701 (16)	0.0829 (19)	0.0738 (18)	0.0131 (14)	0.0119 (14)	0.0102 (14)
C9	0.135 (3)	0.081 (2)	0.153 (4)	0.027 (2)	0.041 (3)	−0.012 (2)
C10	0.0527 (11)	0.0668 (14)	0.0620 (14)	0.0011 (11)	0.0006 (10)	−0.0056 (12)
C11	0.0817 (19)	0.111 (3)	0.106 (2)	0.0086 (19)	0.0401 (18)	0.022 (2)
C12	0.090 (2)	0.151 (4)	0.135 (3)	0.003 (2)	0.051 (2)	−0.023 (3)
C6	0.101 (2)	0.139 (3)	0.085 (2)	−0.013 (2)	0.012 (2)	0.042 (2)
N1	0.0544 (9)	0.0391 (9)	0.0470 (10)	0.0028 (7)	−0.0065 (7)	0.0042 (7)
N2	0.0514 (9)	0.0403 (9)	0.0452 (10)	−0.0020 (7)	−0.0070 (7)	−0.0003 (7)
N3	0.0556 (8)	0.0365 (7)	0.0425 (8)	−0.0003 (7)	−0.0068 (7)	0.0030 (8)
S1	0.1045 (5)	0.0372 (3)	0.0596 (4)	−0.0009 (3)	−0.0251 (4)	−0.0006 (3)
C1	0.0522 (10)	0.0395 (10)	0.0435 (11)	0.0010 (8)	−0.0003 (8)	0.0020 (8)
S2	0.0818 (4)	0.0363 (2)	0.0627 (4)	0.0006 (2)	−0.0236 (3)	0.0047 (2)
C2	0.0377 (8)	0.0468 (10)	0.0454 (11)	−0.0006 (8)	0.0006 (7)	0.0032 (9)
S3	0.0603 (3)	0.0634 (3)	0.0624 (4)	0.0002 (3)	−0.0234 (3)	0.0022 (3)
C3	0.0455 (10)	0.0404 (9)	0.0441 (11)	−0.0015 (7)	−0.0009 (9)	0.0029 (8)

### Geometric parameters (Å, °)

**Table:**

N4—C7	1.503 (3)	C10—H10B	0.9700
N4—C10	1.509 (3)	C11—C12	1.474 (5)
N4—C4	1.511 (3)	C11—H11A	0.9700
N4—H4	0.925 (10)	C11—H11B	0.9700
C4—C5	1.506 (4)	C12—H12A	0.9600
C4—H4A	0.9700	C12—H12B	0.9600
C4—H4B	0.9700	C12—H12C	0.9600
C5—C6	1.510 (4)	C6—H6A	0.9600
C5—H5A	0.9700	C6—H6B	0.9600
C5—H5B	0.9700	C6—H6C	0.9600
C7—C8	1.504 (3)	N1—C2	1.360 (3)
C7—H7A	0.9700	N1—C1	1.386 (3)
C7—H7B	0.9700	N1—H1	0.915 (10)
C8—C9	1.499 (5)	N2—C2	1.350 (2)
C8—H8A	0.9700	N2—C3	1.376 (3)
C8—H8B	0.9700	N2—H2	0.908 (10)
C9—H9A	0.9600	N3—C1	1.341 (2)
C9—H9B	0.9600	N3—C3	1.343 (2)
C9—H9C	0.9600	S1—C1	1.674 (2)
C10—C11	1.489 (4)	S2—C3	1.679 (2)
C10—H10A	0.9700	C2—S3	1.6525 (19)
			
C7—N4—C10	112.32 (16)	N4—C10—H10A	108.9
C7—N4—C4	113.42 (18)	C11—C10—H10B	108.9
C10—N4—C4	111.51 (19)	N4—C10—H10B	108.9
C7—N4—H4	109.2 (19)	H10A—C10—H10B	107.7
C10—N4—H4	104.8 (17)	C12—C11—C10	114.8 (3)
C4—N4—H4	104.9 (17)	C12—C11—H11A	108.6
C5—C4—N4	114.92 (18)	C10—C11—H11A	108.6
C5—C4—H4A	108.5	C12—C11—H11B	108.6
N4—C4—H4A	108.5	C10—C11—H11B	108.6
C5—C4—H4B	108.5	H11A—C11—H11B	107.5
N4—C4—H4B	108.5	C11—C12—H12A	109.5
H4A—C4—H4B	107.5	C11—C12—H12B	109.5
C4—C5—C6	110.7 (2)	H12A—C12—H12B	109.5
C4—C5—H5A	109.5	C11—C12—H12C	109.5
C6—C5—H5A	109.5	H12A—C12—H12C	109.5
C4—C5—H5B	109.5	H12B—C12—H12C	109.5
C6—C5—H5B	109.5	C5—C6—H6A	109.5
H5A—C5—H5B	108.1	C5—C6—H6B	109.5
N4—C7—C8	114.8 (2)	H6A—C6—H6B	109.5
N4—C7—H7A	108.6	C5—C6—H6C	109.5
C8—C7—H7A	108.6	H6A—C6—H6C	109.5
N4—C7—H7B	108.6	H6B—C6—H6C	109.5
C8—C7—H7B	108.6	C2—N1—C1	123.70 (17)
H7A—C7—H7B	107.5	C2—N1—H1	119.4 (17)
C9—C8—C7	111.1 (3)	C1—N1—H1	116.6 (17)
C9—C8—H8A	109.4	C2—N2—C3	124.54 (17)
C7—C8—H8A	109.4	C2—N2—H2	115.0 (17)
C9—C8—H8B	109.4	C3—N2—H2	120.4 (17)
C7—C8—H8B	109.4	C1—N3—C3	119.75 (16)
H8A—C8—H8B	108.0	N3—C1—N1	119.04 (17)
C8—C9—H9A	109.5	N3—C1—S1	121.99 (15)
C8—C9—H9B	109.5	N1—C1—S1	118.96 (15)
H9A—C9—H9B	109.5	N2—C2—N1	113.81 (16)
C8—C9—H9C	109.5	N2—C2—S3	123.12 (15)
H9A—C9—H9C	109.5	N1—C2—S3	123.06 (15)
H9B—C9—H9C	109.5	N3—C3—N2	118.95 (17)
C11—C10—N4	113.6 (2)	N3—C3—S2	122.30 (15)
C11—C10—H10A	108.9	N2—C3—S2	118.75 (15)
			
C7—N4—C4—C5	61.7 (3)	C2—N1—C1—N3	2.8 (3)
C10—N4—C4—C5	−66.3 (3)	C2—N1—C1—S1	−176.22 (15)
N4—C4—C5—C6	−172.3 (3)	C3—N2—C2—N1	−3.0 (3)
C10—N4—C7—C8	174.1 (2)	C3—N2—C2—S3	178.48 (15)
C4—N4—C7—C8	46.6 (3)	C1—N1—C2—N2	1.3 (3)
N4—C7—C8—C9	170.0 (3)	C1—N1—C2—S3	179.77 (16)
C7—N4—C10—C11	63.9 (3)	C1—N3—C3—N2	3.6 (3)
C4—N4—C10—C11	−167.5 (2)	C1—N3—C3—S2	−175.92 (16)
N4—C10—C11—C12	−177.1 (3)	C2—N2—C3—N3	0.7 (3)
C3—N3—C1—N1	−5.2 (3)	C2—N2—C3—S2	−179.76 (16)
C3—N3—C1—S1	173.75 (16)		

### Hydrogen-bond geometry (Å, °)

**Table:**

*D*—H···*A*	*D*—H	H···*A*	*D*···*A*	*D*—H···*A*
N4—H4···N3	0.93 (1)	1.95 (1)	2.867 (2)	172 (3)
N1—H1···S2^i^	0.92 (1)	2.51 (1)	3.4037 (17)	167 (2)
N2—H2···S1^ii^	0.91 (1)	2.39 (1)	3.2911 (17)	170 (2)

Symmetry codes: (i) −*x*+1, *y*−1/2, −*z*+3/2; (ii) −*x*+1, *y*+1/2, −*z*+3/2.
